# Chinese classical decoction Wuwei Xiaodu Drink alleviates gout arthritis by suppressing NLRP3-Mediated inflammation

**DOI:** 10.3389/fphar.2024.1388753

**Published:** 2024-07-26

**Authors:** Na Lin, Qiaoding Dai, Yan Zhang, Liping Xu

**Affiliations:** Rheumatology and Immunology, The First Affiliated Hospital of Zhejiang Chinese Medical University (Zhejiang Provincial Hospital of Chinese Medicine), Hangzhou, Zhejiang, China

**Keywords:** Wuwei Xiaodu Drink, gout arthritis, NLRP3 inflammasome, inflammatory cytokines, clinical trail and efficacy

## Abstract

**Background:** Wuwei Xiaodu Drink (WWXDD), a classical decoction of traditional Chinese medicine, has been clinically used for the treatment of gout in China for many years. This study aimed to demonstrate the efficacy of WWXDD in treating gout flares and elucidate its underlying therapeutic mechanism.

**Methods:** A randomized control trial was conducted to compare the effectiveness of WWXDD with low-dose colchicine in gout arthritis. The primary outcome was the clinical response rate on the 7th day, and joint syndrome score and serological tests were secondary outcome measures and were compared in the two groups on the 1st and 7th day. Then we used a network pharmacology approach to investigate the possible mechanism of WWXDD in treating gout, and the effects of WWXDD on the MSU-induced rat model were observed.

**Results:** In the clinical trial, a total of 78 participants completed the study, and the results demonstrated comparable clinical complete response rates, joint symptom scores, and serological test outcomes between the two groups on the 7th day. Network pharmacology analysis identified 51 core genes that target gout and WWXDD interactions. Notably, strong significant correlations were observed with inflammation cytokine genes and metabolism-related genes. Furthermore, it was found that WWXDD reduced gene expression levels of inflammation cytokines including IL-1β, TNF, and IL-18 in an MSU-induced rat model while increasing IL-10 expression. Additionally, WWXDD decreased insulin gene expression in this model. Moreover, WWXDD exhibited a reduction in both gene and protein expressions associated with the NLRP3-mediated inflammatory pathway in inflamed joints of rats.

**Conclusion:** The results of the present study suggested the anti-inflammatory effects of WWXDD in the treatment of gouty arthritis, partially through inhibiting NLRP3 inflammasome activation.

**Clinical Trial Registration:** ClinicalTrials.gov, identifier ChiCTR2100047807.

## Introduction

Gout is a common form of inflammatory arthritis characterized by sudden episodes of intense joint pain and swelling. These symptoms occur due to a persistently high level of uric acid in the blood (hyperuricemia), leading to the accumulation of monosodium urate crystals in joints, tendons, and other tissues (Dalbeth et al., 2021). The global prevalence of gout ranges from 0.68% to 3.9%, with an incidence rate varying between 0.58 and 2.89 per 1,000 person-years worldwide (Schlee et al., 2018; Dehlin et al., 2020; Singh and Gaffo, 2020). Previous epidemiological studies have demonstrated the association of gout with multiple comorbidities such as a cluster of metabolic diseases, coronary heart disease, and kidney failure, which damage human health and attract global attention (Bardin and Richette, 2017; Singh and Gaffo, 2020). Currently, the treatment strategy for gout is mainly uric acid-lowing drugs and anti-inflammatory management, including benzbromarone, febuxostat, allopurinol, non-steroid anti-inflammatory drugs (NSAIDs), corticosteroids, and colchicine. These medications may cause adverse effects on the liver, kidney, or gastrointestinal system, leading to a decrease in patients' adherence to treatment (Dugowson and Gnanashanmugam, 2006; Finkelstein et al., 2010; Kapugi and Cunningham, 2019; Dehlin et al., 2020; Dalbeth et al., 2021). As a result, the physical, emotional, social, and economic burdens of gout are widely acknowledged, and seeking economical, effective, and safe medicines is urgent.

Traditional Chinese medicine (TCM) has been utilized for thousands of years in the prevention and treatment of gout due to its affordability, safety, and effectiveness. Extensive research has confirmed that TCM effectively alleviates joint pain and swelling while reducing levels of erythrocyte sedimentation rate, C-reactive protein, and serum uric acid during gout flares (Liu et al., 2021; Pu et al., 2021). Previous studies also demonstrated that classical TCM formulas and agents isolated from some botanical drugs could reduce inflammatory reactions, regulate cellular immunity, and improve metabolism in the treatment mechanism of gout (Liu et al., 2014; Liang et al., 2021; Xu et al., 2022). Wuwei Xiaodu Drink (WWXDD), a traditional Chinese medicine compound, has been applied to treat gout and achieved a satisfactory effect in China. Modern pharmacological studies demonstrated that it possessed anti-inflammation effects and the regulation of Foxp3 + CD25^+^CD4^+^ Treg cells via the IL-2/STAT5 signaling pathway (Huang et al., 2022). The botanical drugs included in the formula have been scientifically proven to possess uric acid-lowering effects and exhibit inhibitory activity against xanthine oxidase (XOD) (Lee et al., 2017).

Nevertheless, the mechanism of WWXDD against gout is still unclear. In the study, we evaluated the effectiveness and safety of WWXDD in the treatment of gouty arthritis and investigated the potential pharmacological mechanisms by network pharmacology, then further analyzed the expression of inflammation cytokines and key protein NOD-, LRR- and pyrin domain-containing protein 3(NLRP3) expression in MSU-induced arthritis rat model (So and Martinon. 2017).

## Materials and methods

### Preparation of experimental drugs

The combination of WWXDD consisted of *Lonicera japonica Thunb.* (*Caprifoliaceae, Lonicera L.*). (Jin Yin Hua, batch No. 211127) 10 g; *Chrysanthemum indicum L.* (*Compositae, Chrysanthemum L*.) (Ye Ju Hua, batch No. 20220101) 10 g; *Elephantopus scaber L*. (*Compositae, Taraxacum F.H.Wigg*.) (Pu Gong Ying, batch No. 20220101) 10 g; *Viola philippica var. philippica* (*Violaceae, Viola L.*) (Zi Hua Di Ding, batch No. 20210901) 10 g; *Semiaquilegia adoxoides (DC.) Makino* (*Ranunculaceae, Semiaquilegia Makino*) (Tian Kui Zi, batch No. 20211101) 10 g. The herbal materials were obtained from the Zhejiang Provincial Hospital of Chinese Medicine (Zhejiang, China) and subjected to double decoction with eight times the volume of water for 1 h. Subsequently, the extracts were combined, filtered, and concentrated at 100°C to obtain a final volume of 50 mL, corresponding to a concentration of 1 g/mL. Colchicine was obtained from JiaLin Pharmaceutical Co. Ltd. (Beijing, China), specification 0.5 mg/tablet. Monosodium urate (MSU) was purchased from Shanghai Yuanye Biotechnology Co., Ltd.

### Quality control and content determination of WWXDD

The metabolites and quality control of WWXDD were analyzed using high-performance liquid chromatography (HPLC). HPLC analysis was conducted on a Thermo U3000 UHPLC system from Thermo, San Jose, United States. A column named CAPCELL PAK C18 MG II (150 mm × 4.6 mm, 3 μm) manufactured by Osaka Soda in Japan was employed. The mobile phase consisted of acetonitrile (A) and 0.2% formic acid (B), with the elution gradient set as follows: 0–40 min, 5%–29% A, at a flow rate of 0.4 mL/min. To ensure consistency, the column temperature was maintained at 30°C while injecting a volume of 5 μL. For mass spectrometry data acquisition, a Q-Exactive mass spectrometer from Thermo in San Jose, United States was utilized. The specific MS parameters and compound annotation were consistent with previous descriptions (Meng et al., 2023).

### Clinical study design

The clinical study was a single-center, prospective, randomized controlled trial carried out at The First Affiliated Hospital of Zhejiang Chinese Medical University (Zhejiang Provincial Hospital of Chinese Medicine). Subjects diagnosed with gout from August 2021 to January 2022 were identified. This study was approved by the Ethics Committee of the First Affiliated Hospital of Zhejiang Chinese Medical University with identifier 2021-KL-034-01. Written informed consent for study participation was obtained from all patients. This trial protocol is registered at the Chinese Clinical Trial Registry with the registration number of ChiCTR2100047807.

### Inclusion and exclusion criteria

The inclusion criteria were as follows: 1. aged between 18 and 75 years, 2. according to 1977 or 2015 American College of Rheumatology’s (ACR) diagnostic criteria for gout arthritis (Wallace et al., 1977; Neogi et al., 2015), 3. gout flare at admission time, 4. with informed signed consent and voluntary participation in the study, 5. TCM syndrome pattern of heat according to the guidelines for TCM diagnosis and treatment for gout.

The exclusion criteria were as follows: (1) poor compliance, loss to follow-up, drug abusers, (2) stage 4/5 chronic kidney disease (glomerular filtration rate/creatinine clearance<30 mL/min), (3) impaired liver function, either AST or ALT exceeds the upper limit of the reference value by 2 times, (4) recent surgery or gastrointestinal bleed, history of gastric ulcer, (5) previous inability to low-dose colchicine, (6) chronic heart disease, malignant tumors, recurrent infectious diseases, pancytopenia, (7) allergic to various drugs in the protocol, (8) pregnancy or lactation, (9) other inflammatory arthritis that may confuse GA, such as rheumatoid arthritis, ankylosing spondylitis, psoriatic arthritis, systemic lupus erythematosus, Sjogren’s syndrome, and other diseases.

### Randomization and interventions

Participants were randomly allocated 1:1 using number table random sampling to either. Oral WWXDD 25 mL twice a day for 7 days, 2. Oral colchicine 0.5 mg twice a day for 7 days. All patients were required to stop other TCM in the study, and prescribed etoricoxib 60 mg a day. The study was divided into three stages: screening period, treatment period, and safety follow-up period. Participants were screened according to inclusion and exclusion criteria and collected demographic data such as gender, age, height, weight, medication history, joint symptoms score (signal joint symptom was scored on a 0–2 scale:0 no swelling and tenderness, 1 = tenderness, no swelling or swelling, no tenderness, 2 = swelling, and tenderness), count all affected joint, laboratory examination data during the screening period. The joint symptom scores were independently completed by two rheumatologists, and subsequently averaged to obtain the final value. After screening completion, participants who are eligible enter a week treatment period and collect general symptoms, joint symptoms score, and laboratory examination data on day 7. The patients will complete safety follow-up measures 14 days after treatment. Safety evaluation includes the general conditions of the participants, the incidence of adverse events, and laboratory indicators (such as blood cell count, urine routine, stool routine, and hepatorenal function).

### Outcomes

The primary endpoint was the clinical response rate, the clinical remission was defined as follows: (1) complete response: the joint symptoms (swelling and paining) and signs of skin completely disappeared, the joint function returns to normal, serologic inflammatory texts such as C-reactive protein (CRP) returned to normal. (2) partial response: joint symptoms (swelling and paining) and signs of skin alleviated, the joint function improved, or serologic inflammatory texts such as C-reactive protein improved. (3) no remission: the joint symptoms of patients do not change, and joint function did not recover, serologic inflammatory texts such as C-reactive protein were not relieved. Joint symptoms score, and serological texts such as C-reactive protein, and uric acid were made on baseline and 7th day as secondary outcome measures.

### Data collection

Baseline data and outcomes will be collected by a rheumatologist as the above intervention described. An e-mail or message was sent to participants to return on the 7th day and 14th day, if they do not return, a researcher will telephone to capture key outcome data such as joint score, general syndromes, and adverse events.

### Network pharmacology for WWXDD targeted genes

The target genes of WWXDD were retrieved from the BATMAN-TCM database (http://bionet.ncpsb.org/batman-tcm) by inputting the five Chinese botanical drugs using their respective capitalized pinyin names and utilizing default settings to acquire compound composition and forecast target genes. Gout-related genes were obtained by searching for the keyword “gout” in GeneCards and OMIM databases, which were summarized to obtain comprehensive information on disease targets. An R language script was executed to determine overlap between WWXDD-related prediction targets and gout disease targets, visually represented through a VENN diagram. Furthermore, protein interaction network construction was performed by inputting drug-disease intersection genes into STRING online database (http://string-db.org/), with Cytoscape 3.9.0 software used to generate a network diagram illustrating the relationship between WWXDD, active ingredients, gout, and their corresponding targets.

### Animals and preparation of gouty arthritis model

A total of 36 male Sprague-Dawley (SD) rats, weighing about 250 g, were purchased from BK Company in Shanghai and raised in the SPF animal feeding room of the Animal Center of Zhejiang Chinese Medicine University for 1 week at temperature (22°C ± 2°C), humidity (75 ± 5) %, clean drinking water, free feeding, 12 h control day and night. After 7 days of acclimatization, rats were randomly divided into six groups (6 rats in each group): Normal control group (NC), gouty arthritis model group, colchicine treated group (colchicine tablet 0.1 mg/kg), low (2 g/kg), medium (4 g/kg) and high (8 g/kg) dose of WWXDD treated group. 3 days before induction, rats were given the corresponding drug intervention by intragastric administration, and the model and control groups were given the same volume of distilled water once a day for 7 days. Gout arthritis models were induced by injection of 0.2 mL urate crystal suspension into the tarsus planters of the right foot with a 4.5-gauge needle on the fourth day of feeding. Control rats received an injection of 10 μL of PBS.

### Evaluation of joint arthritis

24 h, 48 h, and 72 h after modeling, the rats in each group were evaluated by visually inspecting the degree of joint inflammation, reaction to stimulus and change of weight, and the sum of all degrees. The assessment was conducted based on the following criteria: (1) The severity of arthritis was graded on a scale ranging from 0 to 3 as described below: absence of any signs of redness and swelling received a score of 0; the presence of mild redness and swelling at the midfoot (tarsals) or ankle joint received a score of 1; mild redness and swelling extending from the ankle to the midfoot received a score of 2; mild redness and swelling extending from the ankle to the metatarsal joints received a score of 3. (2) Reaction to the stimulus was scored from 0–3 as follows: 0, no response to stimuli; 1, mild response to stimuli; 2, moderate response to stimuli; 3, serious response to stimuli. (3) Change of weight was scored 0–10 based on percentage weight loss. All these tests were conducted by an investigator (Yan Zhang) who was blinded to the experimental design.

### Histopathological assessment of joint

Rats were killed 72 h after MSU injection, removed right forepaws, 10% formaldehyde solution was fixed, EDTA decalcified for 20 days, and paraffin-embedded. The tissues were then cut to 3 um thickness and conventional morphological evaluation was performed by staining with hematoxylin and eosin (H&E) at a light microscope on ×10 and ×40 objectives. The number of infiltrated inflammatory cells in each observation field of each group was counted using ×40 objectives in a blind manner and normalized with the control group (PBS injected).

### Quantitative real-time PCR (RT-qPCR) and ELISA

The right forepaws of SD rats were used to extract total RNA using TRIzol reagent from Invitrogen, United States. To reverse transcribe the RNA into cDNA, 5× All-In-One RT Master Mix from ABM in Richmond, CA was employed. For real-time PCR analysis, Ultra SYBR Mixture from CWBIO in China and ABI 7500 instrument from ABI in the United States were utilized. Replicate experiments were conducted. The control experiment relied on glyceraldehyde-3-phosphate dehydrogenase (GAPDH) as an internal reference index, and the relative expression level was determined using the 2^−ΔΔCT^ method. The gene primers used in this study were synthesized by Gemma Gene Co., LTD. and their sequences can be found in Table 1. The levels of TNF-α and IL-10 in serum were quantified using an enzyme-linked immunosorbent assay (ELISA) kit (ELK biotechnology, ELK1396 and ELK1144), following the manufacturer’s instructions.

**TABLE 1:** Primers used for reverse transcription-quantitative polymerase chain reaction.

Gene	Primer sequence
*GAPDH*	*F: 5′-GCA​AGT​TCA​ACG​GCA​CAG-3′*
	*R: 5′-GCC​AGT​AGA​CTC​CAC​GAC​AT-3′*
*IL-1*β	*F: 5′-CCA​CAG​ACC​TTC​CAG​GAG​AAT​G-3′*
	*R: 5′-GTG​CAG​TTC​AGT​GAT​CGT​ACA​GG-3′*
*IL-10*	*F: 5′- CAT​GCT​GCT​GGG​CCT​GAA-3′*
	*R: 5′-CGT​CTC​CTT​GAT​CTG​CTT​GAT​G-3′*
*TNF*	*F: 5′- GCT​TGT​CCC​TGC​TAC​CCG​C-3′*
	*R: 5′-GTC​AGG​GGA​TGT​GGC​GTC​T-3′*
*IL-6*	*F: 5′- TAG​TCC​TTC​CTA​CCC​CAA​TTT​CC-3′*
	*R: 5′- TTG​GTC​CTT​AGC​CAC​TCC​TTC-3′*
*INS*	*F: 5′- CCT​GTG​GAT​GCG​CTT​CCT​G-3′*
	*R: 5′-TCC​AGT​TGG​TAG​AGG​GAG​CA-3′*
*APOE*	*F: 5′-GCT​CCC​AAG​TCA​CAC​AGG​AA-3′*
	*R: 5′-TAT​CTG​CTG​GGT​CTG​CTC​CT-3′*
*APOA1*	*F: 5′-GGG​AGT​TCT​GGC​AGC​AAG​AT-3′*
	*R: 5′-TCG​ATC​AGG​GTA​GGG​TGG​TT-3′*
*LEP*	*F: 5′- ATG​TGC​TGG​AGA​CCC​CTG​T-3′*
	*R: 5′- GCA​TTC​AGG​GCT​AAG​GTC​CA-3′*
*Caspase-1*	*F: 5′- GGA​AAC​AAA​AGT​CGG​CAG​AG-3′*
	*R: 5′- ACG​CTG​TAC​CCC​AGA​TTT​TG*
*NLRP3*	*F: 5′- ATG​AAC​TCC​TTC​TCC​ACA​AGC​GCC​TTC-3′*
	*R: 5′-TGG​CTT​GTT​CCT​CAC​TAC​TCT​CAA​ATC-3′*
*Pro-IL-1β*	*F:5′- CAC​GAT​GCA​CCT​GTA​CGA​TCA-3′*
	*R: 5′- GTT​GCT​CCA​TAT​CCT​GTC​CCT-3′*
*IL-18*	*F: 5′-ATG GCT GCC ATG TCA GAA GA-3′*
	*R: 5′- TTG TTA AGC TTA TAA ATC ATG CGG CCT CAG G-3′*

### Tissue collecting and Western blotting analysis

The right forepaws were collected 72 h after the injection of MSU. They were then weighed and frozen rapidly in liquid nitrogen. Tissues were homogenized using a Bullet Blender (BBX24, Next Advance Inc. NY, United States) at full speed for 20 min at 4°C in a solution containing 50-mM Tris-base (pH 7.4), 150-mM NaCl, protease inhibitors (Roche, Switzerland), and 0.2% Triton X-100 (Sigma, MO, United States). The resulting mixture was centrifuged at 13,500× g for 12 min at 4°C to obtain the supernatant. Protein concentrations were determined using the BCA assay kit from Thermo Fisher (MA, United States). Subsequently, these samples underwent separation through electrophoresis on a gel with a concentration of SDS-PAGE of 10%. The proteins were then transferred onto a PVDF membrane via electrophoretic transfer method. To block non-specific binding sites on the membranes, they were incubated with skim milk diluted to a concentration of 5% for 1 h. Following this step, primary antibodies including NLRP3 (1:1000), IL-1β (1:1000), Caspase1 (1:1000), IL-18 (1:1000) and internal control GAPDH (1:1000) from Abcam (Cambridge MA., United States) were added to the membranes and allowed to incubate overnight at a temperature of four degrees Celsius. Afterward, horseradish peroxidase-conjugated secondary antibody was applied followed by detection using chemiluminescence reagents known as ECL obtained from Bio-Rad company. Digital scanning was performed to capture images of the membranes and AzueSpot analysis software was utilized for quantification purposes regarding signal intensity present in these digital images.

### Statistical analysis

We utilized SPSS 24.0 software for data processing and analysis, and GraphPad Prism 9 for analysis and drawing figures. Measurement data were presented as case numbers, mean, standard deviation, minimum, median, maximum, upper quartile (Q1), and lower quartile (Q3), with a significance level of *p* < 0.05. Continuous variables following normal distribution were reported as mean ± SD, whereas non-normal distribution parameters were expressed as median (Q1–Q3). Dichotomous data were represented by case numbers (percentage of total) and analyzed using the χ2 test; statistical significance was determined at *p* < 0.05. For pairwise comparisons between groups with equal variances, t-tests were utilized for independent sample group comparisons; in cases of unequal variances, Kruskal–Wallis H tests were applied instead. To assess differences among multiple group comparisons, a one-way analysis of variance (ANOVA) was conducted.

The statistical analysis of clinical trials involved the utilization of both the full analysis set (FAS) and per protocol (PP) set analyses on the case population. The FAS analysis encompassed all randomized cases that received drug treatment at least once. In instances where the complete treatment course could not be observed, missing data was imputed using the last observation carried forward method to ensure robustness of final results, while an intention-to-treat analysis was conducted for efficacy and adverse reactions. On the other hand, the PP set consisted of cases that met inclusion criteria, adhered well to the treatment plan without taking prohibited drugs, and successfully fulfilled all requirements specified by the case report form.

## Results

### Quantitative analysis of chemical constituents of WWXDD

The Supplementary Material presents the characteristic chromatograms of both the standard and WWXDD. Supplementary Figure S1A displays the base peak chromatogram (BPC) of the WWXDD sample, while Supplementary Figure S1B shows the HPLC-UV chromatogram at 280 nm. To determine the precise molecular mass, we utilized Xcalibur Software to analyze additional ions in negative ion mode, considering a mass tolerance of 5.0 ppm and isotope abundance. Through preliminary identification of their retention time, precise molecular weight, and chemical structures as well as the in-house library searching, and previous literature, we found a total of 89 metabolites such as chlorogenic acid, aesculetin, luteolin, and loniceroside A (Supplementary Table S1).

### Efficacies of WWXDD on gout flares

**FIGURE 1:**
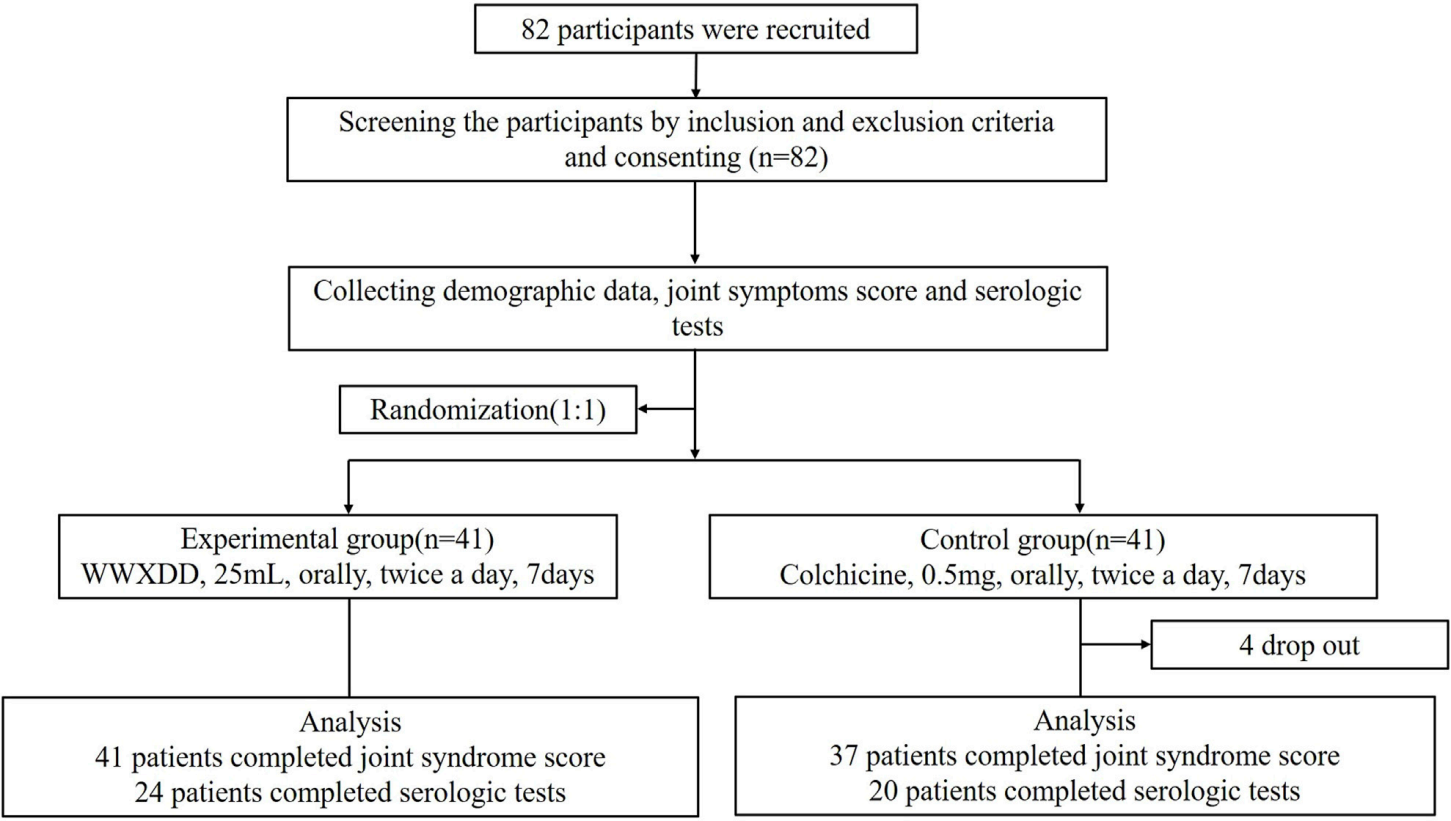
Flow chart of clinical study participants. A total of 82 adults with gout flares were enrolled and randomly assigned to receive either WWXDD (25 mL, twice per day) or a low-dose of colchicine (0.5 mg twice per day) for a duration of 7 days. Among them, primary outcome data was completed by 78 participants (WWXDD *n* = 41, colchicine *n* = 37), while serologic tests at day 7 were completed by 44 participants (WWXDD *n* = 24, colchicine *n* = 20).

A total of 82 patients were recruited and randomized, with 78 completing primary outcome data. Among them, 41 patients received WWXDD treatment while 37 received colchicine treatment. Additionally, serologic tests were completed by 44 participants on day 7, including 24 in the WWXDD group and 20 in the colchicine group. Figure 1 illustrates a flow chart depicting the study procedure. Table 2 demonstrates that there were no significant differences between the two groups in terms of age, sex, first instance of gout, number and location of affected body parts, joint symptoms score, BMI, baseline levels of CRP, uric acid, alanine transaminase (ALT), aspartate aminotransferase (AST), or creatinine at baseline.

**TABLE 2:** Comparison of the clinical features and serological parameters of the both trails at baseline.

Characteristics		WWXDD	Colchicine	Difference
Baseline				*P*
Age: mean (SD)		49.85 (15.85)	51.8 (19.6)	0.561
Male, n (%)		39 (95.12%)	34 (91.89%)	0.628
BMI: mean (SD)		25.21 (2.44)	26.99 (3.85)	0.051
First instance of gout, n (%)		7 (17.07%)	6 (16.22%)	0.92
Tophus, n (%)		12 (29.27%)	13 (35.14%)	0.579
Number of body parts affected, n (%)	1	20 (48.78%)	22 (59.56%)	0.779
	2	13 (31.71%)	5 (13.51%)	
	3	5 (12.20%)	5 (13.51%)	
	4	2 (4.88%)	1 (2.70%)	
	≧5	1 (2.44%)	4 (10.81%)	
Body part affected, n (%)	metatarsophalangeal joint (MTPJ)	12 (29.27%)	14 (37.84%)	0.423
	foot joints	28 (68.29%)	19 (51.35%)	0.127
	Other lower limb	13 (31.71%)	12 (32.43%)	0.945
	upper limb	14 (34.15%)	12 (32.43%)	0.873
Joint symptoms score, Q2 (Q1-Q3)		2 (2–4)	2 (2–4.5)	0.266
CRP mg/L, mean (SD)		37.11 (32.20)	27.33 (38.57)	0.133
Uric Acid (μmol/L), mean (SD)		494.63 (151.67)	490.54 (134.19)	0.90
ALT (U/L), mean (SD)		31.05 (20.11)	38.29 (40.87)	0.34
AST (U/L), mean (SD)		30.84 (33.29)	28.37 (27.46)	0.73
Cr (μmol/L), mean (SD)		88.10 (19.12)	81.41 (22.96)	0.28

MTPJ: metatarsophalangeal joint; CRP: C-reactive protein; ALT: alanine transaminase; AST: aspartate aminotransferase; Cr: Creatinine; SD: standard deviation.

**FIGURE 2:**
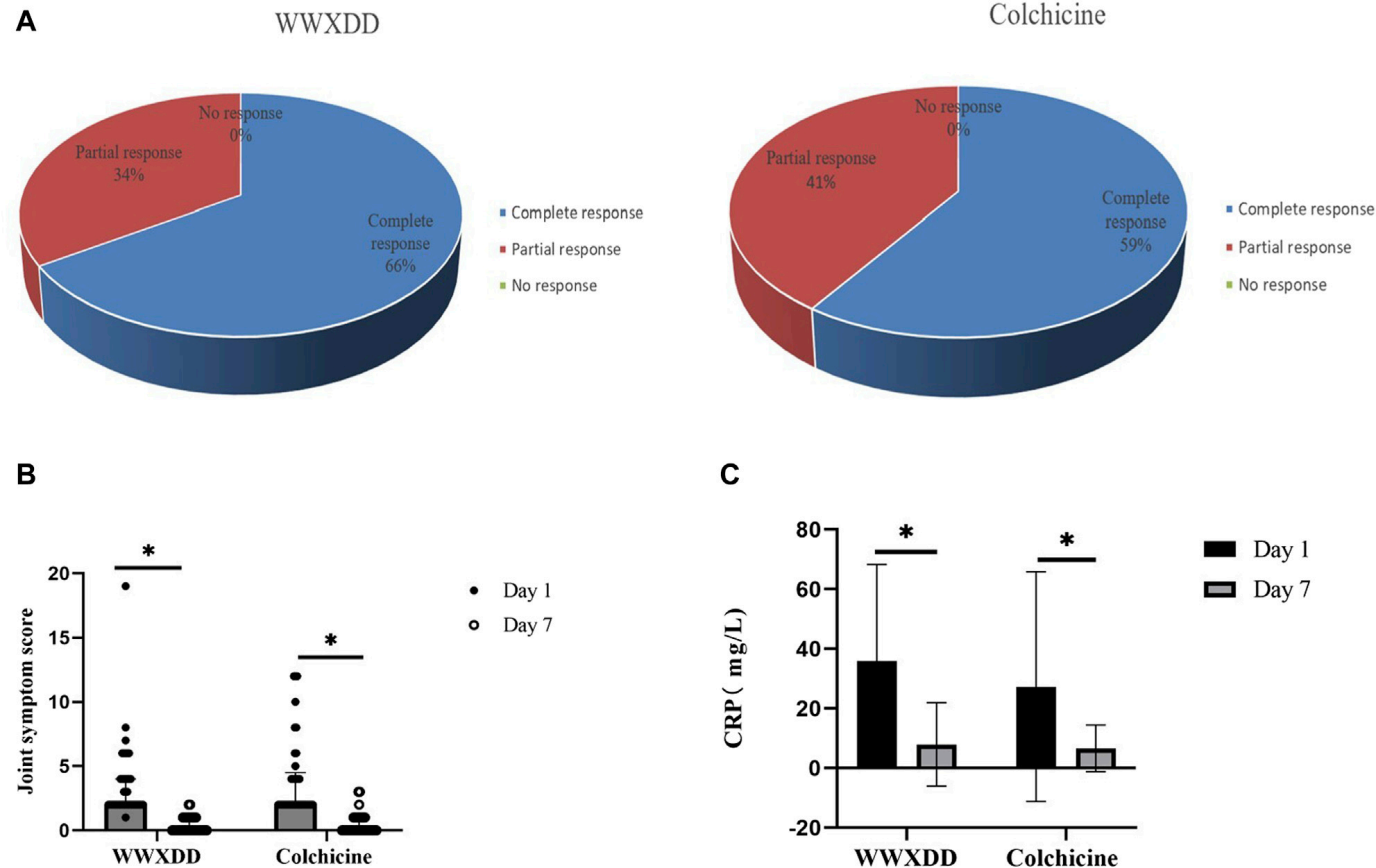
Efficacies of WWXDD on gout flare patients. **(A)** All patients achieved clinical remission in both groups. The complete response rate was 66% in the WWXDD group and 59% in the colchicine group, which showed no significant difference between the two groups (*p* = 0.642). **(B)** There were no significant differences in joint symptom scores on the 7th day between the two groups (*p* = 0.539). However, compared with the scores on the 1st day, there were significant improvements observed in both groups (WWXDD: 1st day vs. 7th day, 2(2-4) vs 0(0-1), *p* < 0.001; Colchicine: 1st day vs. 7th day, 2 (2–4.5) vs. 0 (0–1), *p* < 0.001). **(C)** Levels of C-reactive protein on the 7th day did not differ significantly between the WWXDD and colchicine groups (WWXDD vs colchicine, mean ± SD: 7.96 ± 13.97 vs. 6.65 ± 7.81, *p* =0 .71). However, when compared with levels on the first day, there were significant reductions observed in both groups (WWXDD: 1st day vs. 7th day, mean ± SD: 37 .11 ± 32 .20 vs. 7 .96 ± 13 .97, *p* = 0 .004; Colchicine: 1st day vs. 7th day, mean ± SD: 27 .33 ± 38 .57 vs. 6 .65 ± 7 .81, *p* = 0.008). WWXDD, WWXDD group; Colchicine, positive control group. * *p* < 0.05.

Four patients withdrew from the study without providing a reason, all of whom were in the colchicine group. The complete response rate was 66% in the WWXDD group and 53.55% in the colchicine group, with no statistically significant difference observed between these two groups as determined by FAS analysis (*p* = 0.103). By PP set, comparable outcomes were demonstrated between the WWXDD group (complete response rate of 66%) and colchicine group (complete response rate of 59%, *p* = 0.642; Figure 2A). Additionally, a comparison of joint symptom scores and serological tests on the first and seventh day revealed no significant differences between the two groups regarding joint symptom scores, uric acid levels, or CRP at day seven (Table 3).

**TABLE 3:** Comparison of outcome measures at day 7 in both groups.

Outcome measures	WWXDD				Colchicine				P	
	Baseline	Day 7	Mean difference (95%CI)	P _intra_	Baseline	Day 7	Mean difference (95%CI)	P _intra_	Mean difference (95%CI)	P _day 7_
Joint symptoms score, Q2 (Q1-Q3)	2 (2–4)	0 (0–1)		<0.001	2 (2–4.5)	0 (0–1)		<0.001		0.539
CRP mg/L, mean (SD)	37.11 (32.20)	7.96 (13.97)	28.66 (14.00–43.32)	0.004	27.33 (38.57)	6.65 (7.81)	29.32 (7.93–50.71)	0.008	−0.9 (-21.90–6.47)	0.71
Uric Acid (μmol/L), mean (SD)	494.63 (151.67)	437.89 (139.61)	56.74 (-15.16–128.64)	0.120	490.54 (134.19)	452.30 (116.42)	38.24 (-29.66–106.13)	0.26	14.41 (-58.95–87.77)	0.70
ALT (U/L), mean (SD)	31.05 (20.11)	29.63 (13.31)	1.42 (-7.29–10.14)	0.745	38.29 (40.87)	42.25 (39.7)	−4.0 (-26.71–18.78)	0.73	12.62 (-3.69–28.93)	0.126
AST (U/L), mean (SD)	30.84 (33.29)	26.33 (18.86)	4.51 (-9.74–18.75)	0.530	28.37 (27.46)	26.20 (19.60)	2.17 (-11.84–16.19)	0.76	−0.13 (-11.53–11.26)	0.98
Cr (μmol/L), mean (SD)	88.10 (19.12)	91.36 (22.69)	−3.27 (-14.83–128.64)	0.745	81.41 (22.96)	83.65 (22.74)	−2.24 (-15.18–10.70)	0.73	−7.7 (-21.90–6.47)	0.29

CRP: C-reactive protein; ALT: alanine transaminase; AST: aspartate aminotransferase; Cr: Creatinine; SD: standard deviation.

Both the WWXDD group and the colchicine group exhibited significant within-group improvements in joint symptom scores and C-reactive protein (CRP) levels on days 1 and 7. Specifically, regarding joint symptoms, the results demonstrated that patients in the WWXDD group had a median score of 2 (interquartile range [IQR]: 2–4) on day 1 compared to a median score of 0 (IQR: 0–1) on day 7, with a statistically significant difference (*p* < 0.001). Similarly, patients in the colchicine group showed a median score of 2 (IQR: 2–4.5) on day 1 versus a median score of 0 (IQR: 0–1) on day 7, also indicating a significant improvement (*p* < 0.001) (Figure 2B). The levels of CRP demonstrated a significant decrease compared to the baseline of treatment (WWXDD: 1st day vs. 7th day, 37.11 ± 32.20 vs. 7.96 ± 13.97, mean difference (95% CI): 28.66 (14.00–43.32), *p* = 0.004; Colchicine: 1st day vs. 7th day, 27.33 ± 38 .57 vs. 6 .65 ± 7 .81, mean difference (95% CI):29 .32 (7 .93–50 .71), *p* = 0.008). (Table 3; Figure 2C).

No serious adverse events were observed during the trials. Moreover, one participant who received WWXDD reported diarrhea on day 5 and was advised to discontinue the medication. Fortunately, his symptoms subsided within a day without requiring any further intervention. Both treatment groups exhibited normal hepatorenal function before and after therapy (Table 3).

### Network pharmacology showed the Venn diagram, intersection gene, protein interaction, and WWXDD-target-gout visual regulatory network

**FIGURE 3:**
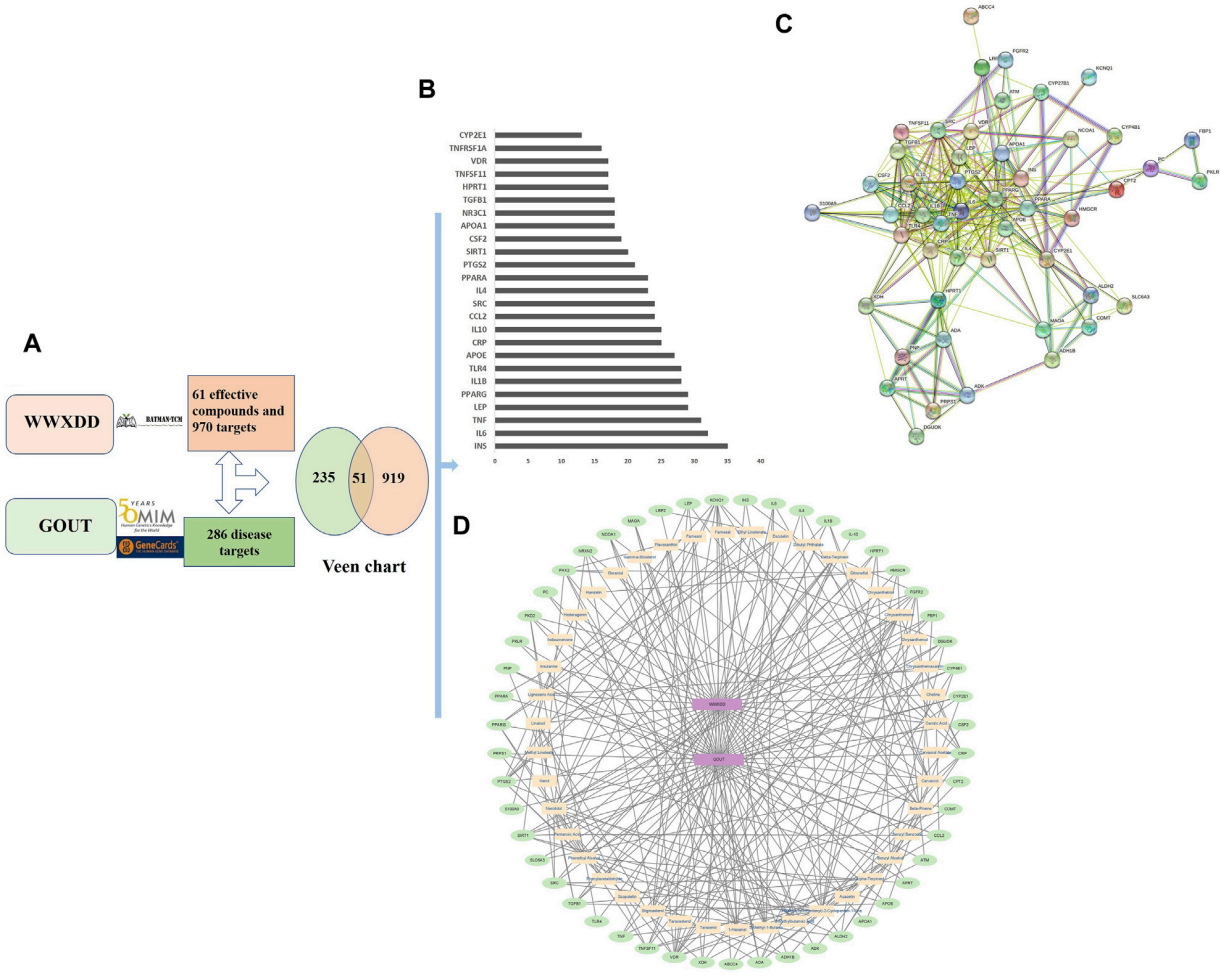
Network pharmacology showed Venn diagram, intersection gene, protein interaction and WWXDD-target-gout visual regulatory network. **(A)** A total of 61 effective compounds and 970 gene action targets of WWXDD were obtained through the BATMAN-TCM platform, while 286 related genes of gout were selected from GeneCards and OMIM databases. By intersecting these two sets of targets, a final set of 51 targets for the intersection between WWXDD and gout disease was identified. **(B)** The core target gene diagram revealed 26 core target genes with higher-than-average degree values. Notably, inflammatory factors IL-6, TNF-α, IL-1β, TLR4, and IL-10 exhibited degree values greater than twice the average value. Additionally, metabolism-related genes such as INS, LEP, APOE also displayed higher degrees. **(C)** The protein interaction network diagram depicted a network consisting of 51 protein nodes connected by 313 interaction relationships (i.e., edges), with three protein nodes lacking any interaction relationship. The average local clustering coefficient was calculated to be 0.683 while the average degree was found to be 12.3. **(D)** The visual regulatory network illustrated the connections between target genes (represented by elliptical nodes on the left side) and effective compounds (represented by elliptical nodes on the right side). This intricate connection reflects multiple pathways through which various effective compounds in WWXDD act upon target genes.

A total of 98 effective compounds were predicted through BATMAN-TCM platform retrieval, of which 61 were active components, and 970 gene action targets were obtained. A total of 1549 gout disease targets were selected through GeneCards and OMIM databases, and 286 related genes with relevant source scores greater than or equal to 1 were selected. By taking the intersection of the two targets, a total of 51 targets of the intersection of WWXDD and gout disease were finally obtained (Figure 3A). 26 core target genes were screened according to the greater-than-average degree value, in which inflammatory factor tumor necrosis factor (TNF), interleukin (IL) −6, IL-1β, toll-like receptor (TLR) 4 and IL-10, and metabolism-related genes insulin (INS), leptin (LEP), Apolipoprotein (APO) E, et al., were greater than twice the average degree value (Figure 3B). The intergene-protein interaction network of the intersecting target was constructed by the STRING online database. The protein interaction network contains 51 protein nodes and 313 interaction relationships (i.e., the connection lines between nodes), and 3 protein nodes with no interaction relationship. The average avg. local clustering coefficient is 0.683. The average degree is 12.3 (Figure 3C). The visual regulatory network of target-Gout disease was developed using Cytoscape 3.9.0 software components. There were 51 elliptical nodes on the left for target genes and 45 elliptical nodes on the right for effective compounds. The intricate connection reflects a variety of effective compounds in WWXDD and multiple pathways to act on target genes. (Figure 3D).

### WWXDD alleviates MSU-induced arthritis in rats

**FIGURE 4:**
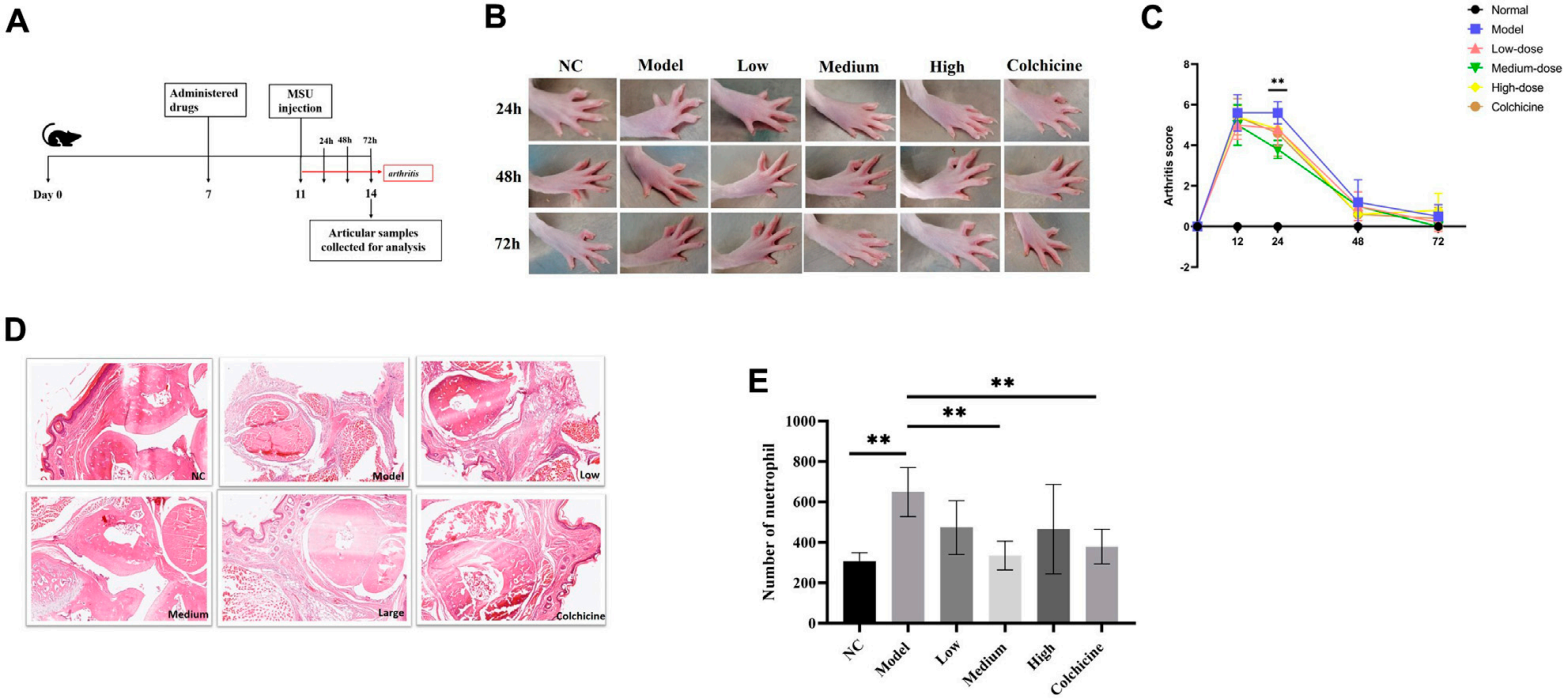
WWXDD alleviates MSU-induced arthritis in rats. **(A)** Timetable of drug administration and MSU induction in rats: Rats were treated with WWXDD or colchicine on day 7, followed by injection of MSU into the right forepaw on day 11. Arthritis severity scores were recorded every 24 h after MSU induction. **(B)** Representative photographs of each rat group were taken at 24 h, 48 h, and 72 h. **(C)** Arthritis severity scores were evaluated and compared every 24 h after MSU induction. The results showed a significant decrease in arthritis score in the medium dose of WWXDD group compared to the model group at 24 h (*p* <0.01). **(D)** Representative microscopic images of H&E stained paws from each group are shown (magnifications: ×100). **(E)** Summarized data revealed a significant reduction in the number of infiltrated neutrophils per observation field in inflamed joint tissues for both the medium dose of WWXDD and colchicine groups compared to the model group (*p* <0.01). NC, normal control group; Model, model group; Low, low dose WWXDD group; Medium, medium dose WWXDD group; High, high dose WWXDD group; Colchicine, positive control group. Results are derived from 3 separate experiments. **p < 0.01, *p < 0.05.

The effects of WWXDD on MUS-induced arthritis were also investigated in rats. A flow chart is shown in Figure 4A. The results showed that after 24 h of MSU injection, the arthritis score of the medium of the WWXDD group was significantly decreased compared with the model group (*p* < 0.01) (Figures 4B, C). Then the effects of WWXDD on inflammatory cell infiltration induced by MSU in induced joints were examined. As shown in Figure 4D, joint tissues in the model group showed marked infiltration of inflammatory cells and hyperplasia of the synovial membrane than the normal control group, and a medium dose of WWXDD and colchicine groups showed the infiltration of inflammatory cells and hyperplasia of the synovial membrane were inhibited. Meanwhile, the effects of WWXDD on neutrophil infiltration were examined in per observation field (400 X), as shown in Figure 4E, compared with the model group, the count of neutrophils infiltration in medium dose of WWXDD and colchicine groups was significantly decreased in inflamed joint tissues (*p* < 0.01).

### WWXDD suppressed the expression of pro-inflammatory cytokines, INS, and promoted the expression of interleukin-10

**FIGURE 5:**
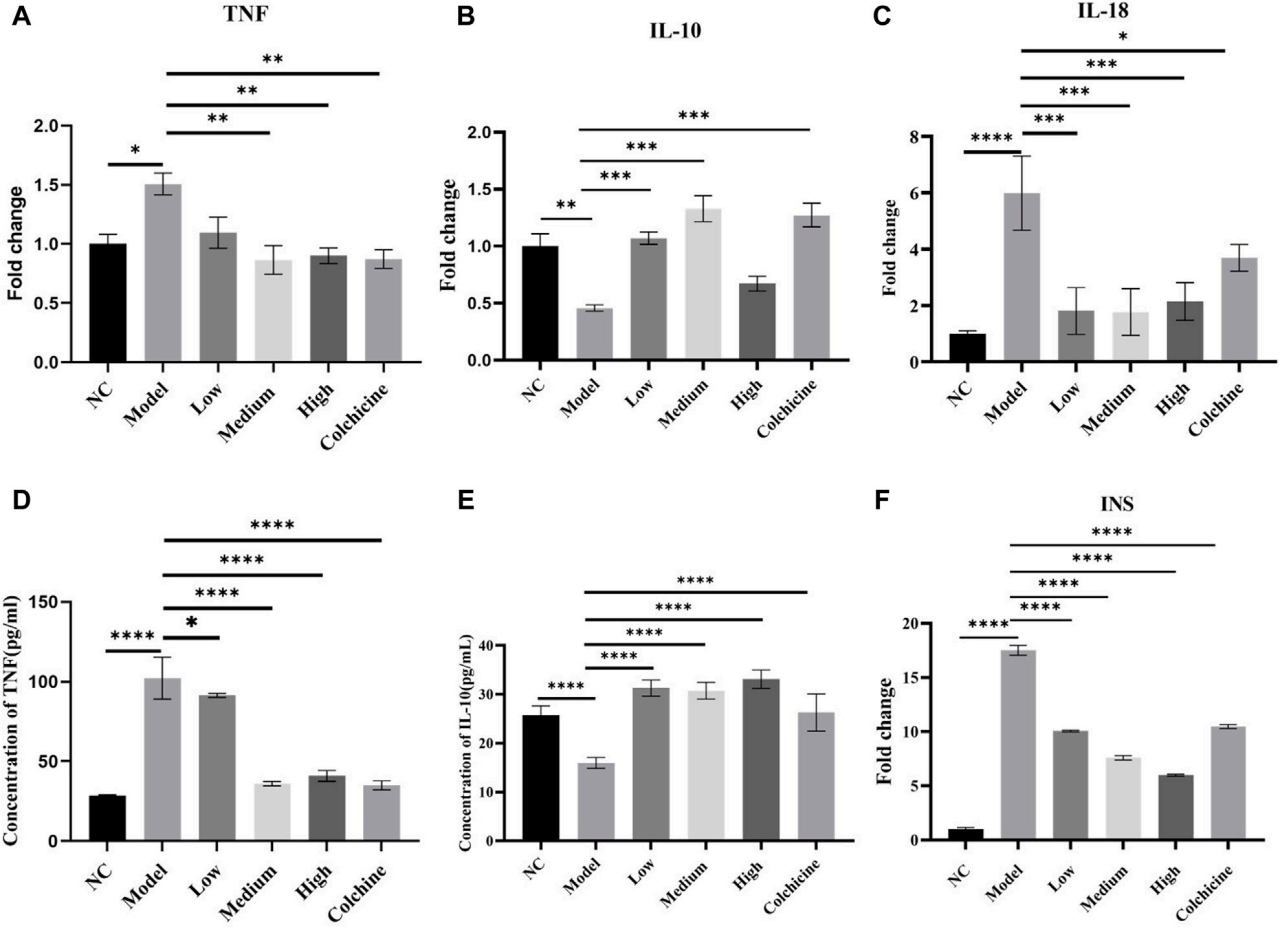
WWXDD suppressed the expression of pro-inflammatory cytokines, INS, and promoted the expression of interleukin-10. **(A)** The mRNA expression of TNF in the model group (1.507 ± 0.159) was significantly higher than that in the control group (1 ± 0.141), while it was significantly decreased in the WWXDD medium-dose (0.865 ± 0.208), high-dose (0.9 ± 0.113), and colchicine groups (0.873 ± 0.136) (*p*< 0.01). **(B)** The mRNA expression level of IL-10 was significantly lower in the model group (0.46 ± 0.05) compared to the control group (1.00 ± 0 .19). Conversely, it exhibited significant increases in both low-dose WWXDD treatment group (1 .07± 0.10), medium-dose WWXDD treatment group (1.33 ± 0.20), and colchicine treatment group (1.27 ±0.18) (*p* < 001). **(C)** The mRNA expression of IL-18 in the model group (5.9 ± 1.32) was significantly higher compared to that in the control group (1.0 ± 0.11), whereas it was significantly reduced in the WWXDD low-dose (1.81 ± 0.83), medium-dose (1.77 ± 0.83), high-dose (2.15 ± 0.67), and colchicine groups (3.70 ± 0.47) (P< 0.01). **(D)** The serum concentration of TNF (pg/ml) in the model group (102.21 ± 13.14) was significantly elevated compared to that in the control group (28.51 ± 0.57), whereas WWXDD low-dose (91.41 ±1.23), medium-dose (36.09 ± 1.34), and high-dose groups (40.90 ± 3.35), as well as the colchicine group (34.99 ± 2.77), exhibited a substantial reduction relative to the model group (*p* < 0.05). **(E)** The serum concentration of IL-10 (pg/ml) in the model group (15.97 ± 1.11) was significantly lower compared to that in the control group (25.72 ± 1.89), whereas WWXDD low (31.27 ± 1.64), medium (30.72 ± 1.72), and high (33.09 ± 1.88) dose groups as well as the colchicine group (26.29 ± 3.80) exhibited a significant increase when compared to the model group (*p* < 0.001).**(F)** The mRNA expression of INS in the model group (17.51 ± 0.45) exhibited a significant increase compared to the control group (1.0 ± 0.11). Conversely, there was a notable decrease in the expression of WWXDD in the low dose group (10.06 ± 0.09), medium dose group (7.59 ± 0.19), high dose group (5.98± 0.009), and colchicine group (10.48 ± 0.16) with statistical significance observed (*p* < 0.01). NC, normal control group; Model, model group; Low, low dose WWXDD group; Medium, medium dose WWXDD group; High, high dose WWXDD group; Colchicine, positive control group. All data were shown as mean ± SEM. Results are derived from 3 separate experiments. **p < 0.01, ***p < 0.001, ****p <0.0001.

The results presented in Figure 5 demonstrate a significant upregulation of TNF expression in both joint tissues and serum of the model group compared to the normal control group (*p* < 0.01). In contrast, there was a notable reduction in IL-10 expression when compared to the normal control group (*p* < 0.01). Compared to the model group, TNF mRNA expression exhibited a significant decrease in the medium-dose, high-dose WWXDD, and colchicine groups (*p* < 0.01) (Figure 5A), accompanied by a corresponding reduction in serum concentration in the low, medium, high-dose, and colchicine groups (*p* < 0.05) (Figure 5D). Notably, joint tissues treated with low or medium doses of WWXDD and colchicine demonstrated an upregulation of IL-10 mRNA expression (*p* < 0.01) (Figure 5B), which was also observed in serum concentration for all dose levels and the colchicine group (*p* < 0.001) (Figure 5E). The mRNA level of IL-18 in the model group exhibited a significant increase compared to that in the normal group, whereas the expression of IL-18 was significantly reduced in the low-dose, medium-dose, and high-dose WWXD groups as well as the colchicine groups when compared to the model group. (*p* < 0.05) (Figure 5C) However, there was no significant statistical difference in protein expression levels of IL-18 (Supplementary Figure S2). Regarding INS expression levels, they were found to be significantly higher in the model group compared to the control group; however, treatment with low-dose WWXDD, medium-dose WWXDD, high-dose WWXDD, and colchicine led to a substantial decrease in INS expression levels (*p* < 0.01) (Figure 5F). No significant differences were observed among all groups for IL-6, LEP APOA1, and APOE expressions (Supplementary Figure S2).

### WWXDD reduced the expression of NLRP3-mediated inflammatory pathways

**FIGURE 6:**
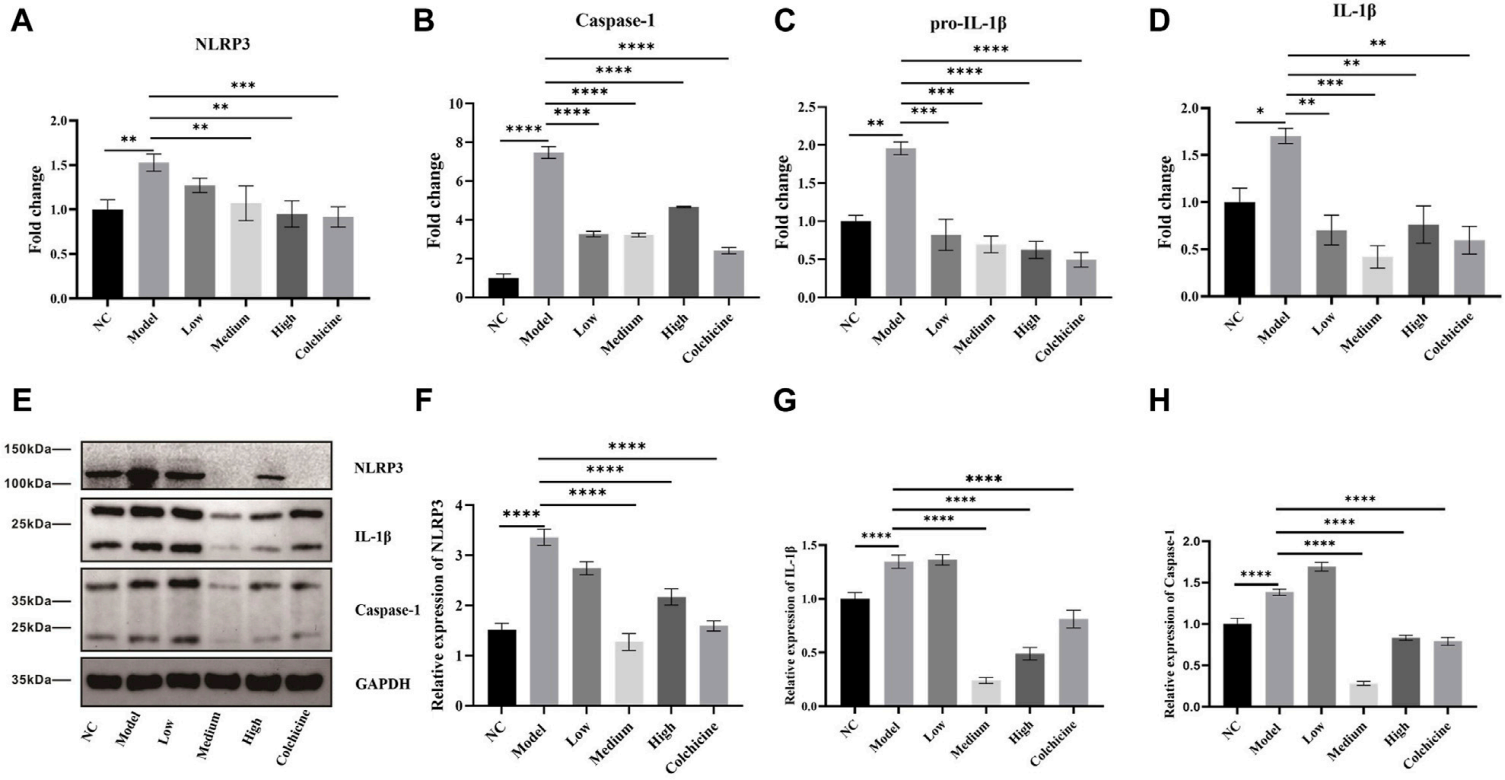
WWXDD reduced the expression of NLRP3-mediated inflammatory pathways. **(A)** The mRNA expression of NLRP3 in the model group (1.53 ± 0.097) was significantly higher than that in the control group (1 ± 0.11), and the expression of NLRP3 in the WWXDD medium-dose (1.07± 0.20), high-dose (0.95 ± 0.15), and colchicine groups (0.92 ± 0.11) showed a significant decrease (*p* < 0.01). **(B)** Caspase-1 mRNA expression in the model group (7.48 ± 0.53) was significantly higher than that in the control group (1.00 ± 0.37). However, there was a significant decrease in caspase-1 expression observed in the WWXDD low-dose (3.27 ± 0.24), medium-dose (3.21 ± 0.16), high-dose (4.66 ± 0.06) and colchicine group (2.41 ± 0.27) compared to the model group (*p* < 0.01). **(C)** Pro-IL-1β mRNA expression levels were significantly elevated in the model group (1.96 ± 0.14) compared to those of control group (1.0 ± 0.13). Conversely, treatment with WWXDD at low dose (0.82 ± 0.36), medium-dose (0.70 ± 0.19), high-dose (0.63 ± 0.19) or colchicine (0.50 ± 0.17) resulted in a significant reduction of pro-IL-lβ gene expression (*p* < 0.01). **(D)** The mRNA expression level of IL-lβ was markedly increased in the model group (1.70 ± 0.14) as compared to that of control group (1.0 ± 0.46). In contrast, administration of WWXDD at low dose low-dose (0.70 ± 0.28), medium-dose (0.42 ± 0.21), high-dose (0.76 ± 0.34), or colchicine (0.60 ± 0.26) led to a substantial decrease m IL-lβ gene expression (P < 0.01). **(E)** Western blot analysis revealed distinct bands for NLRP3, Caspase-I, and IL-l β proteins following treatment with WWXDD. **(F–H)** Quantitative analysis demonstrated reduced protein expressions for NLRP3, caspase-I, and IL-l β after administration of WWXDD (*p* < 0.01). NC, normal control group; Model, model group; Low, low dose WWXDD group; Medium, medium dose WWXDD group; High, high dose WWXDD group; Colchicine, positive control group. All data were shown as mean ± SEM. Results are derived from 3 separate experiments. **p < 0.01, ***p < 0.001, ****p <0.0001.

NLRP3 inflammasome activation plays a critical role in the development of gout arthritis, leading to the activation of caspase-1 and facilitating the proteolytic cleavage and maturation of pro-IL-1β into biologically active IL-1β (So and Martinon. 2017; Dalbeth et al., 2021). Subsequently, we quantified the expression levels of cytokines involved in the NLRP3 inflammasome-mediated inflammatory pathway using RT-PCR. Our findings depicted in Figures 6A–D demonstrate a significant upregulation (*p* < 0.01) in mRNA expression levels of NLRP3, caspase-1, pro-IL-1β, and IL-1β within joint tissues from the model group compared to the normal control group. However, treatment with low, medium, and high-dose WWXDD as well as colchicine resulted in a notable downregulation (*p* < 0.01) in mRNA expression levels of caspase-1, pro-IL-1β, and IL-1β when compared to the model group. Additionally, NLRP3 expression was significantly decreased in medium and high-dose WWXDD groups as well as colchicine groups within joint tissues (*p* < 0.01). Furthermore, protein expression levels of NLRP3, caspase-1, and IL-1β were significantly increased (*p* < 0 .001) in joint tissues from the model group compared to the normal control group as shown in Figures 6E–H. However, treatment with WWXDD (medium and high dose groups) as well as colchicine groups led to a decreased protein expression of NLRP3, caspase-1, and IL-1β in joint tissues (*p* < 0 .001) when compared to the model group.

## Discussion

We found obvious within-group improvements in joint symptom scores and C-reactive protein in both groups but no statistically significant difference between WWXDD and the low-dose colchicine group in this study. The complete response rate and adverse effects rate in both groups were comparable. Low-dose colchicine has been proven to be effective and tolerable as NSAIDs in therapy of gout flare (Roddy et al., 2020). Our findings suggest that WWXDD should have equivalent efficacy to low-dose colchicine in gouty arthritis, which should be considered in treating gout arthritis. Previous clinical studies have also confirmed that WWXDD could effectively relieve acute joint pain, reduce swelling, and improve joint function in gout arthritis, which was consistent with our findings (Chi et al., 2020).

Then the network pharmacology method was used to construct the network of WWXDD and gout from the direction of multi-gene targets, and the Veen diagram was drawn. 26 core target genes were screened according to the greater-than-average value, among which the targets greater than twice the average value included inflammatory factor-related genes such as TNF, IL-6, IL-1β, TLR4 and IL-10 et al., metabolism related genes such as insulin, leptin, and apolipoprotein E. It showed WWXDD effectively reduced MUS-induced arthritis and neutrophil infiltration in joint tissues, inhibited pro-inflammatory cytokines IL-1β, TNF, and IL-18 expression in joint tissues of MUS-induced gout arthritis rats, and increased the expression of anti-inflammatory cytokine IL-10, meanwhile, the expression of INS was also obviously decreased. It has also been reported that the elevation of anti-inflammatory cytokine IL-10 may be involved in the self-regulation of acute inflammatory immunity in gout patients (Chen et al., 2011). In terms of metabolism-related genes, we found that WWXDD can inhibit the expression of insulin, while the relationship between uric acid metabolism and insulin is not clear. These suggest that WWXDD exhibits inhibitory effects on the expression of inflammatory factors to alleviate gouty arthritis and the relevance of the purine metabolism pathway is unclear.

The release of IL-1β, IL-18 and TNF are tightly associated with the pathogenesis of gout. The activation of NLRP3 inflammasome is widely recognized to induce caspase-1-dependent release, leading to the secretion and activation of IL-18 and IL-1β. Sustained IL-1β secretion promotes the release of pro-inflammatory cytokines such as other interleukin-like factors and TNF. Additionally, those cytokines recruit monocytes and neutrophils to sites of tissue insults (Desai et al., 2017; So and Martinon. 2017; So et al., 2018). Our results demonstrate that WWXDD inhibits NLRP3 inflammasome activation while reducing caspase-1 expression along with both pro-IL-1β and IL-1β levels. Furthermore, our findings indicate that WWXDD exerts inhibitory effects on the expression of the IL-18 gene in inflamed joints; however, no discernible impact was observed at the protein level. These results suggest that WWXDD attenuates NLRP3 inflammasome activation effectively, making it a promising candidate for clinical application. This research simultaneously verifies the efficacy of WWXDD treatment in clinical settings as well as *in vivo* models by elucidating its mechanism for treating gouty arthritis.

However, this study also has some limitations. Firstly, the pharmaceutical metabolites of the formula are various, and it remains unclear whether the metabolites have antagonistic or synergistic anti-inflammatory properties. Therefore, further *in vitro* experimentation is required to elucidate the specific substance-mediated upstream and downstream mechanisms of NLRP3 inflammasome activation. Additionally, our clinical trial demonstrated a significant improvement in the joint symptoms score of patients treated with WWXDD. However, further investigation is warranted to elucidate the potential multi-target anti-inflammatory mechanism of this drug and explore its underlying mechanisms in detail. Lastly, network pharmacology analysis suggested a potential metabolic regulatory mechanism for WWXDD; however, more research is required to confirm its effect in hyperuricemia models.

## Conclusion

Our research has provided evidence showing that WWXDD attenuates pro-inflammation cytokine production, upregulates anti-inflammation cytokine IL-10, inhibits NLRP3 inflammasome activation, and has a significant treatment effect on gout arthritis, and is worthy of clinical application.

## Data Availability

The original contributions presented in the study are included in the article/Supplementary Material, further inquiries can be directed to the corresponding author.
