# Supplementary material for: Chinese classical decoction Wuwei Xiaodu Drink alleviates gout arthritis by suppressing NLRP3-Mediated inflammation
Source: Front Pharmacol. 2024 Jul 26;15:1388753. doi: 10.3389/fphar.2024.1388753 (PMC11310048; doi:10.3389/fphar.2024.1388753)
Supplement: Supplementary file 1 [file DataSheet1.pdf]

Supplementary Material

Supplementary Figures and Tables

1.1 Supplementary Figures

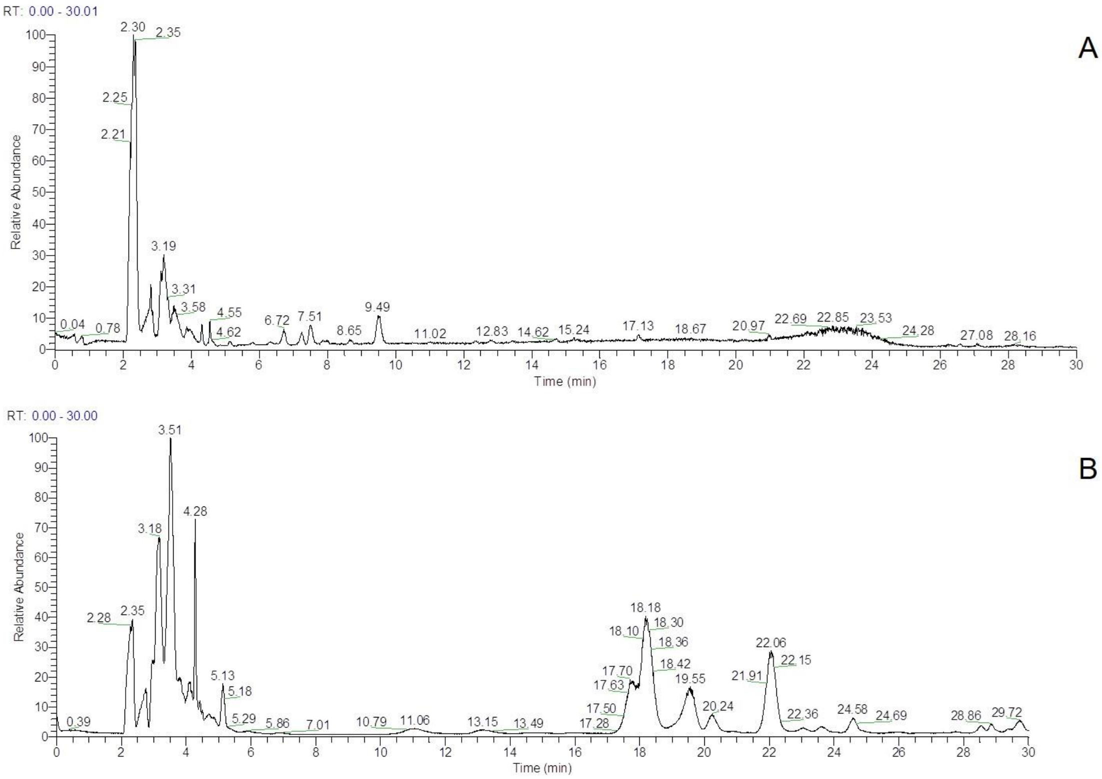

Supplementary Figure 1 HPLC analysis of WWXDD: The BPC of WWXDD in positive (A) and negative (B) mode.

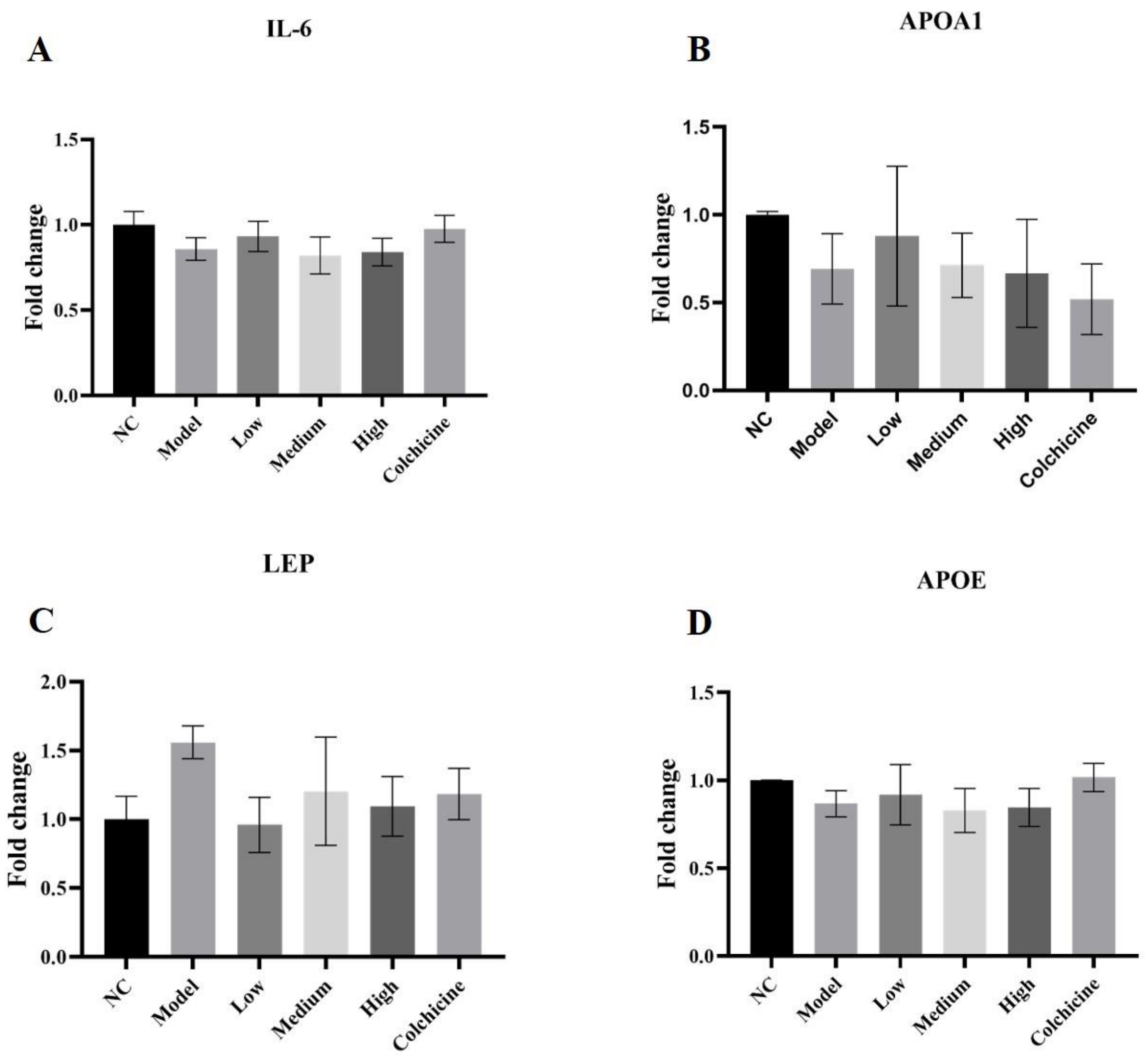

Supplementary Figure 2 Expression of WWXDD on core target genes in inflamed joint tissues of MUS-induced arthritis rats by RT-PCR. There was no significant difference in the expression of IL-6, APOA1, APOE, and LEP among all groups.

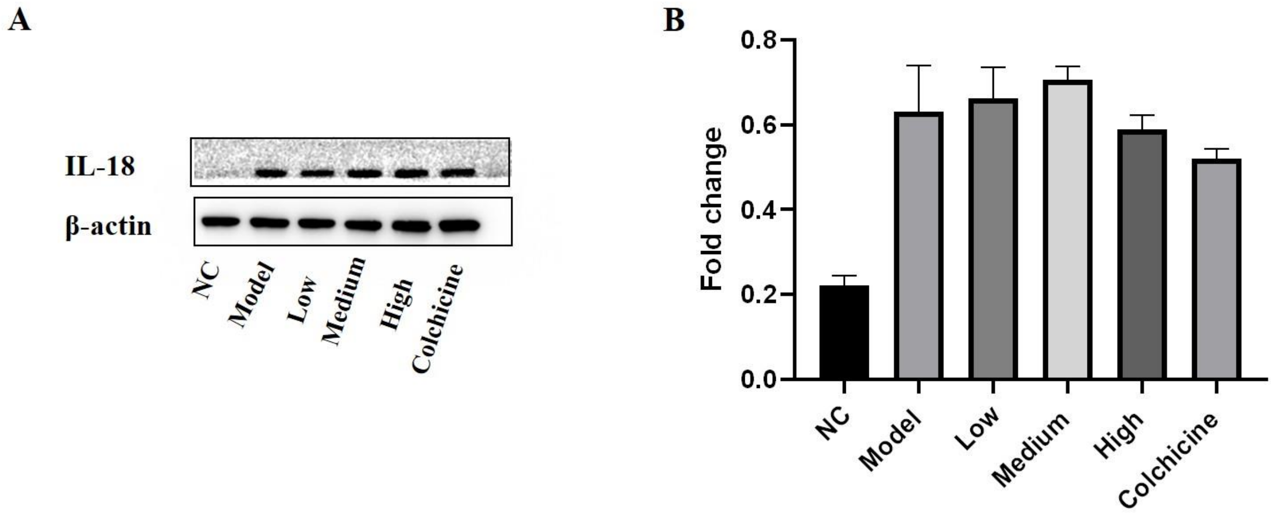

Supplementary Figure 3 Expression of WWXDD on IL-18 protein expression in inflamed joint tissues of MUS-induced arthritis rats. The findings revealed that there was no substantial variation in the levels of IL-18 expression among the Model, WWXDD, and Colchicine groups.

1.2 Supplementary table

Table 1: Mass spectrometry information of chemical components in Wuwei Xiaodu Drink

No.	Name	RT (min)	Formula	Ion Type	Molecular Ion (*m/z*)	Structure
1	3,4,5-Tricaffeoylquinic acid	14.667	$C_{34}H_{30}O_{15}$	$[M+H]^+$		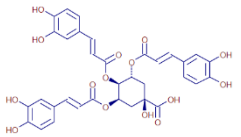
2	3,4-O-Dicaffeoyl quinic acid methyl ester	17.19	$C_{26}H_{26}O_{12}$	$[M-H]^-$	179.0551	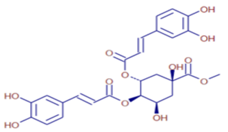
3	3-O-Caffeoylshikimic acid	7.467	$C_{16}H_{16}O_8$	$[M+H]^+$	375.1271	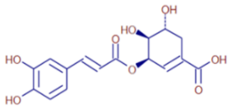
				$[M-H]^-$	373.1134	
4	3,4-dicaffeoylquinic acid	14.672	$C_{25}H_{24}O_{12}$	$[M+H]^+$	183.0862	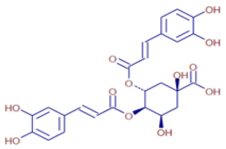
5	4-Hydroxybenzoic acid	8.966	$C_7H_6O_3$	$[M+H]^+$	343.1227	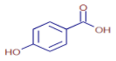
				$[M-H]^-$	341.1083	
6	4-Hydroxycinnamic acid	9.466	$C_9H_8O_3$	$[M+H]^+$	159.0512	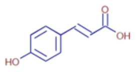
7	5-O-Coumaroylcaffeoylquinic acid	16.753	$C_{25}H_{24}O_{11}$	$[M+H]^+$	193.0706	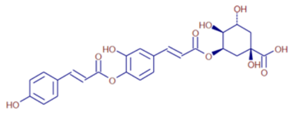
8	5-p-Coumaroylquinic acid	9.521	$C_{16}H_{18}O_8$	$[M+H]^+$	139.0389	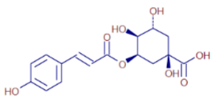
9	6,6-dimethyl-bicyclic [3.1.1] hept-2-ene-2-ethanol.	20.998	$C_{11}H_{18}O$	$[M+H]^+$	110.0604	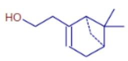
10	7-epi-Vogeloside	10.699	$C_{17}H_{24}O_{10}$	$[M+H]^+$	193.0343	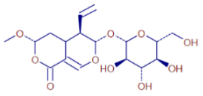

11	7-O-Ethylsweroside	7.423	$C_{18}H_{26}O_{10}$	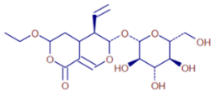
12	7-hydroxy-8-methoxycoumarin	13.919	$C_{10}H_8O_4$	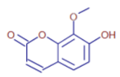
13	8-Epi-loganic acid	8.094	$C_{16}H_{24}O_{10}$	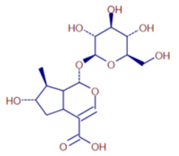
14	Adenosine	3.341	$C_{10}H_{13}N_5O_4$	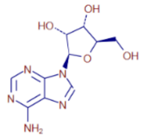
15	Apigenin	20.976	$C_{15}H_{10}O_5$	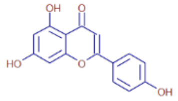
16	Apigenin 6-C-alpha-L-arabinopyranoside-8-C-beta-D-glucopyranoside	6.797	$C_{26}H_{28}O_{16}$	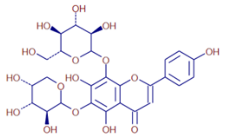
17	Artemitin	9.364	$C_{20}H_{20}O_8$	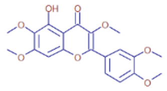
18	Butylidene phthalide	22.113	$C_{12}H_{12}O_2$	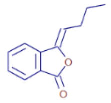
19	Caffeic acid	9.652	$C_9H_8O_4$	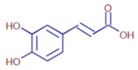

20	Centauroside	13.46	$C_{34}H_{46}O_{19}$	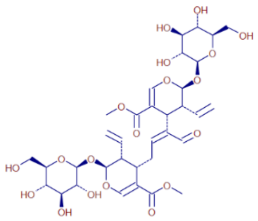
21	Chlorogenic acid butyl ester	5.141	$C_{20}H_{26}O_{9}$	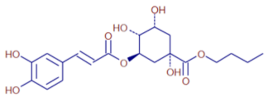
22	Chrysin	13.457	$C_{15}H_{10}O_{4}$	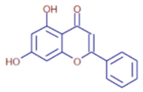
23	Chrysoeriol	21.185	$C_{16}H_{12}O_{6}$	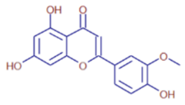
24	Chrysoeriol 7-O-glucopyranoside	14.824	$C_{22}H_{22}O_{11}$	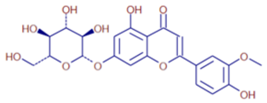
25	Corymbosin	7.497	$C_{19}H_{18}O_{7}$	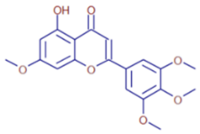
26	Dihydrocaffeic acid	20.837	$C_{9}H_{10}O_{4}$	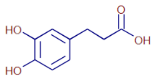
27	Ehretioside	6.934	$C_{14}H_{17}NO_{7}$	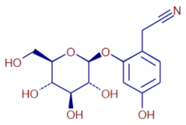

28	Eriodictyol	13.727	$C_{15}H_{12}O_6$	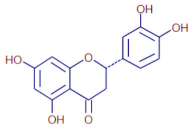
29	Ethyl caffeate	11.225	$C_{11}H_{12}O_4$	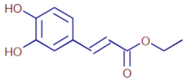
30	Flavoyadorinin B	19.426	$C_{23}H_{24}O_{11}$	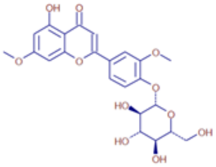
31	Glucose	3.132	$C_6H_{12}O_6$	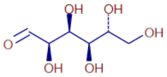
32	Guanosine	3.532	$C_{10}H_{13}N_5O_5$	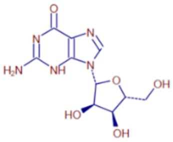
33	Hederagenin	17.967	$C_{30}H_{48}O_4$	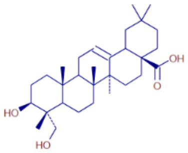
34	Isorhamentin 3-O-rutinoside	7.93	$C_{28}H_{32}O_{16}$	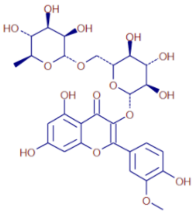

35	Isorhamnetin 3-O-glucopyranoside	16.468	$C_{22}H_{22}O_{12}$	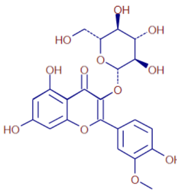
36	Kaempferol 3-O-rutinoside	7.675	$C_{27}H_{30}O_{15}$	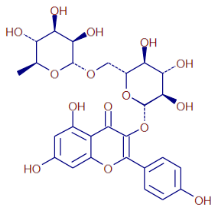
37	Kaempferol-3-O-rhamnoside	14.541	$C_{21}H_{20}O_{10}$	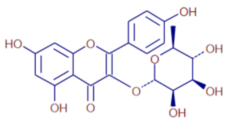
38	Loganin	7.838	$C_{17}H_{26}O_{10}$	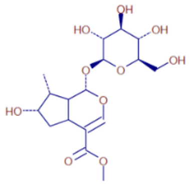
39	Lonfuranacid A	16.464	$C_{12}H_{20}O_{5}$	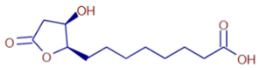
40	Loniceracetalide A	16.813	$C_{21}H_{32}O_{11}$	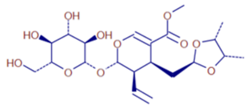
41	Loniceroside A	17.967	$C_{52}H_{84}O_{21}$	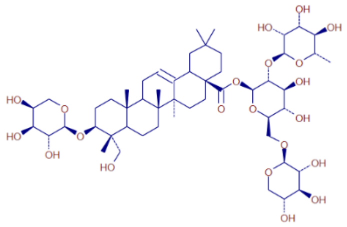

42	Loniceroside B	17.565	$C_{58}H_{94}O_{25}$	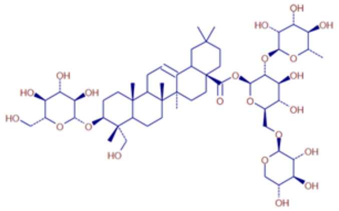
43	Loniceroside C	17.028	$C_{53}H_{86}O_{22}$	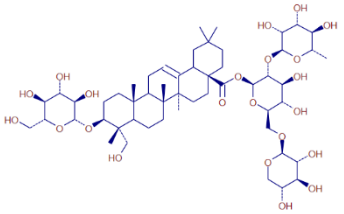
44	Lonijaposide E	3.108	$C_{25}H_{32}NO_{11}$	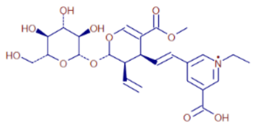
45	Macranthoside A	17.964	$C_{47}H_{76}O_{17}$	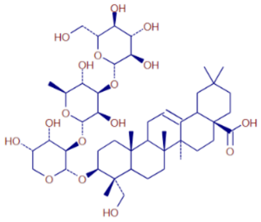
46	Methyl 4-hydroxycinnamate	5.141	$C_{10}H_{10}O_3$	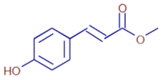
47	Neochlorogenic acid methyl ester	10.239	$C_{17}H_{20}O_9$	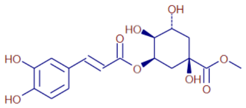
48	Prunetin	17.143	$C_{16}H_{12}O_5$	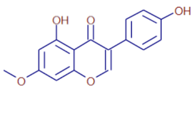

49	Quercetin 3-O-glucopyranoside	13.727	$C_{21}H_{20}O_{12}$	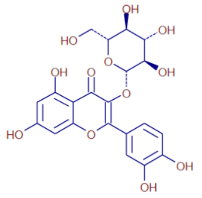
50	Rhoifolin	13.247	$C_{27}H_{30}O_{14}$	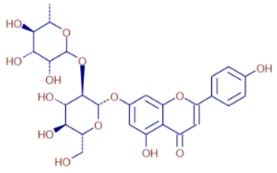
51	Rutin	6.727	$C_{27}H_{30}O_{16}$	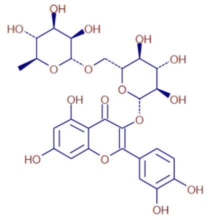
52	Secologanic acid	7.251	$C_{16}H_{22}O_{10}$	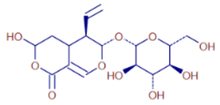
53	Secologanoside	4.533	$C_{16}H_{22}O_{11}$	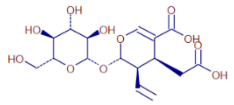
54	Secoxyloganin	7.646	$C_{17}H_{24}O_{11}$	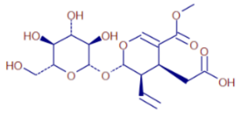
55	Sucrose	3.58	$C_{12}H_{22}O_{11}$	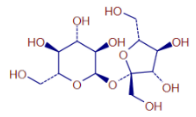

56	Sweroside	8.665	$C_{16}H_{22}O_9$	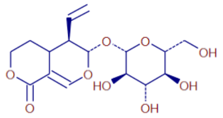
57	syringic acid	4.902	$C_9H_{10}O_5$	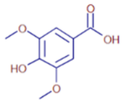
58	Syringin	7.822	$C_{17}H_{24}O_9$	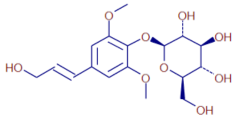
59	thalictricoside	5.507	$C_{19}H_{27}NO_{12}$	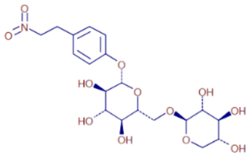
60	trans-Cinnamic acid	7.25	$C_9H_8O_2$	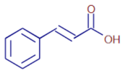
61	Tricin	21.331	$C_{17}H_{14}O_7$	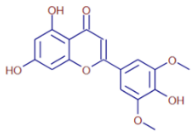
62	Tricin 7-O-glucoside	15.551	$C_{23}H_{24}O_{12}$	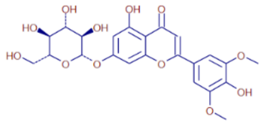
63	Vanillic acid	6.727	$C_8H_8O_4$	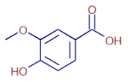
64	Vanillic acid 4-O-β-d-(6-O-benzoylglucopyranoside)	14.942	$C_{21}H_{22}O_{10}$	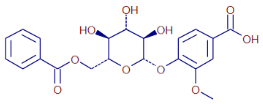

65	Eugenol	17.531	$C_{10}H_{12}O_2$	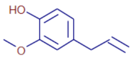
66	Protocatechuic acid	5.596	$C_7H_6O_4$	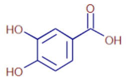
67	Quinate	3.566	$C_7H_{12}O_6$	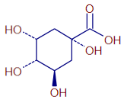
68	Hydroxyphenylacetic Acid	6.496	$C_8H_8O_3$	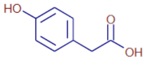
69	Ethyl P-Hydroxyphenylacetate	19.205	$C_{10}H_{12}O_3$	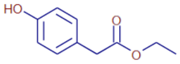
70	Isorhamnetin	19.223	$C_{16}H_{12}O_7$	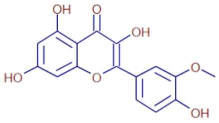
71	Luteolin	19.163	$C_{15}H_{10}O_6$	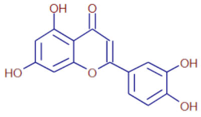
72	Elemicin	8.184	$C_{12}H_{16}O_3$	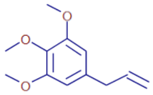
73	Quercetin	12.385	$C_{15}H_{10}O_7$	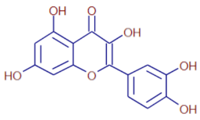

74	Gallic Acid	7.806	$C_7H_6O_5$	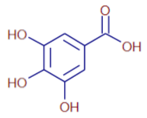
75	Aesculetin	9.512	$C_9H_6O_4$	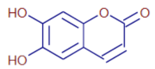
76	Aesculin	6.494	$C_{15}H_{16}O_9$	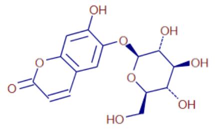
77	Chlorogenic Acid	7.494	$C_{16}H_{18}O_9$	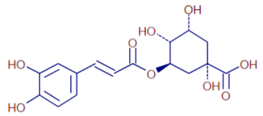
78	Phenylacetic Acid	5.144	$C_8H_8O_2$	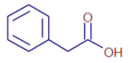
79	Orientin	12.323	$C_{21}H_{20}O_{11}$	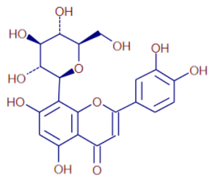
80	Ligustilide	8.184	$C_{12}H_{14}O_2$	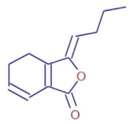
81	Ferulic acid	7.247	$C_{10}H_{10}O_4$	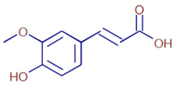

82	Bis（2-ethylhexyl）phthalate	28.137	$C_{24}H_{38}O_4$	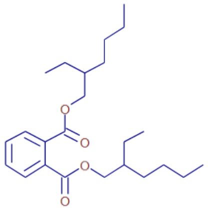
83	p-Hydroxyphenethylalcohol	22.795	$C_8H_{10}O_2$	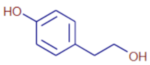
84	7α-Morroniside	4.53	$C_{17}H_{26}O_{11}$	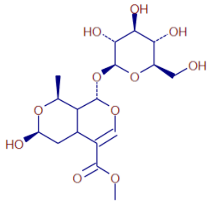
85	Lonijaposide B	4.507	$C_{25}H_{32}NO_{12}$	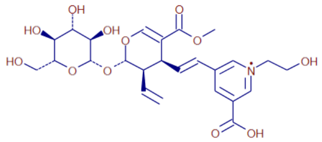
86	Lonijaposide D	20.21	$C_{26}H_{32}NO_{13}$	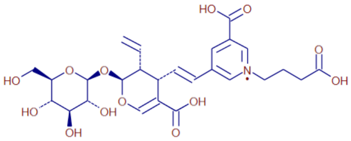
87	Lonijaposide F	19.059	$C_{24}H_{30}NO_{11}$	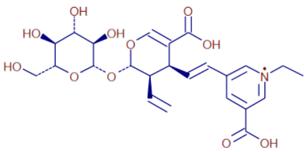
88	Secologanin dimethyl acetal	17.833	$C_{19}H_{30}O_{11}$	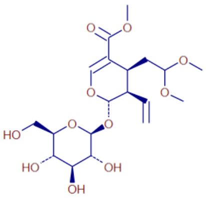

89	Diacetin	16.809	$C_7H_{12}O_5$	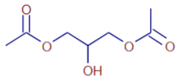
